# Model Calibration for a Rigid Hexapod-Based End-Effector with Integrated Force Sensors

**DOI:** 10.3390/s21103537

**Published:** 2021-05-19

**Authors:** Christian Friedrich, Steffen Ihlenfeldt

**Affiliations:** Chair of Machine Tools and Adaptive Controls, Institute of Mechatronic Engineering, TU Dresden, Helmholtzstrasse 7a, 01069 Dresden, Germany; steffen.ihlenfeldt@tu-dresden.de

**Keywords:** integrated force sensors, sensor networks, calibration and identification, parallel robots

## Abstract

Integrated single-axis force sensors allow an accurate and cost-efficient force measurement in 6 degrees of freedom for hexapod structures and kinematics. Depending on the sensor placement, the measurement is affected by internal forces that need to be compensated for by a measurement model. Since the parameters of the model can change during machine usage, a fast and easy calibration procedure is requested. This work studies parameter identification procedures for force measurement models on the example of a rigid hexapod-based end-effector. First, measurement and identification models are presented and parameter sensitivities are analysed. Next, two excitation strategies are applied and discussed: identification from quasi-static poses and identification from accelerated continuous trajectories. Both poses and trajectories are optimized by different criteria and evaluated in comparison. Finally, the procedures are validated by experimental studies with reference payloads. In conclusion, both strategies allow accurate parameter identification within a fast procedure in an operational machine state.

## 1. Introduction

In production, many manufacturing applications, such as process diagnosis and quality assurance, or adaptive process control for milling, grinding or thermo-smoothing, require in-process force measurement, where, in particular, the measurement of spatial forces and moments in 6 degrees of freedom (DoF) is requested [1]. As one approach, Refs. [1,2,3] present a new measuring system using a hexapod-based end-effector with structure-integrated sensors. Six 1 DoF force sensors are integrated into a rigid bar framework and the measured forces are transformed to Cartesian forces and moments at the tool centre point (TCP) by a control-integrated model.

Compared to commercial 6 DoF force/torque transducers (FT sensors), which are mounted close to the TCP, the new measuring system does not reduce usable workspace, causes no restrictions to spindle mounting and is also less expensive. Compared to a drive current based force measurement in spindle drives, it is far more accurate (factor 100 smaller uncertainty [2]) and requires less modelling effort, since no non-linear and stochastic mechanical influences of drive components interfere such as friction, stick-slip effects or elastic deformation. Altogether, structure-integrated force measurement has many benefits and can complement or replace the measurement by the use of conventional FT sensors as long as it provides a 6 DoF measurement, fulfils accuracy and stiffness requirements, and is simple to parametrise [1]. In addition, integrated sensors can be supplied by the machine manufacturer and make force measurement and control an easy-to-use machine-integrated feature in the future. However, to reach this point, further studies and evidence regarding the parametrisation are required, as hardly any suitable literature on parameter identification for force measurement models is available.

Machine-integrated force measurement solutions have been studied since the beginning of force measurement and are outlined in a detailed state-of-the-art overview in [1]. In short, individual strain-gauge based solutions with a specific design, such as spindles [4], end-effectors [5], or even the workpiece [6], are cost-intensive and do not, thus far, provide a spatial measurement in 6 DoF. Integrated torque sensors in joints [7] still require large mechanical and modelling efforts (friction, stick-slip) and are not advantageous for the desired application in parallel structures and kinematics, where mainly longitudinal forces appear. Concerning the use of rigid Stewart or hexapod structures as force sensors, the most promising works with practical results are a multicomponent calibration system [8,9], a sensor for heavy duty applications [10,11,12], miniaturised sensors, for example, for surgery [13,14], and a measuring system for industrial applications [15]. Still, all of these sensors have been designed as pure (machine independent) measuring devices with static calibration. Neither a movement of the hexapod struts nor of the complete sensor as part of a machine is intended. Consequently, no kinematic or dynamic influences need to be respected within a measurement model.

Instead, for the presented moving sensor system that is the end-effector of a parallel kinematic machine tool, a real-time capable measurement model, which runs within the numeric control system, is essential. As shown in [1,2], an accurate and precise spatial force measurement and competitive characteristics (resolution, range, overload), when compared to a commercial FT sensor, can be realised in the entire workspace by the use of a rigid body dynamic, a sensor and an error compensation model. The stiffness loss at the TCP by sensor integration is comparable to a commercial FT sensor, as presented in [3].

Nevertheless, some of the model parameters, such as mass, centre of gravity and inertia, can change during machine usage, for example through a workpiece or tool change, and need to be re-calibrated. These parameters shall be found using identification procedures, which are the focus of this paper. Referring to parameter identification, many works exist for the identification of rigid body dynamics for serial kinematics [16,17,18,19] and some for parallel kinematics [20,21]. A general overview of identification in robotics is given by the major books [22,23,24]. Where the known works identify only robot, load, and friction parameters, this application requires additional parameters of the sensor and the error model to be identified. In conclusion, the identification of measurement model parameters for parallel structures and kinematics with integrated force sensors improves the potential of the sensor integration approach and is still a novel field of research.

As a new contribution, this work presents a one-step calibration procedure that allows the parametrisation of the measurement models first presented in [1], which are used in the new approach of structure-integrated force measurement in parallel kinematic machine tools. Different model parts, namely dynamic, sensor and error model parameters, are included in the procedure and the feasibility of a fast and easy parameter identification in an operational machine state is demonstrated. In detail, the procedure can run without an experimental setup or special qualification in a few seconds during auxiliary process times, as it is requested in production. Furthermore, the calibration procedure is realised by the use of two different excitation approaches and is proven valid by the identification of reference payloads. The results are applicable for all Stewart Platform based force sensors presented in the literature overview that can now also be applied at the moving end-effector of a machine tool or robot. Finally, FT sensors can also benefit from a measurement model and a parameter identification procedure when used during movement.

## 2. Approach

**Figure 1:**
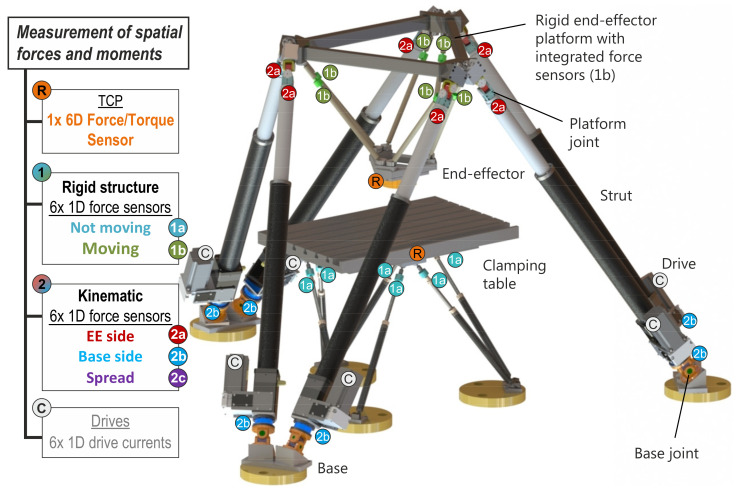
Force sensor integration into hexapod structures and kinematics: approach and sensor placement; 1a: Rigid not moving hexapod structure (e.g., clamping table), 1b: Rigid moving hexapod structure (e.g., end-effector platform), 2x: Hexapod kinematics with sensors in the struts at various positions [1,2,3].

A hexapod-based end-effector is one of multiple options to integrate force sensors into hexapod structures and kinematics, which can be classified into not moving and moving rigid sensor frameworks as well as kinematic-integrated sensors. Figure 1 shows 5 approaches for sensor integration and 2 standard solutions (R: 6 DoF FT sensor, C: drive measurement) as reference. To achieve an accurate measurement, machine and process induced influences need to be compensated from force sensor signals for all setups. Depending on the exact sensor placement, different influences are included into a measurement model, that accordingly requires parameters and online-calculated machine states. The larger the distance between TCP and sensors within the machine structure, the more parameters need to be taken into account, which, as a result, lead to higher modelling and parametrisation efforts, and higher measurement uncertainties. A proof of concept for all setups evaluating quasi-static measurements is presented in [2], which shows models, classification, parameter identification, sensitivity analysis, and experimental results. A more detailed view of the end-effector, including the dynamic model as well as accuracy and precision analyses during quasi-static, dynamic, and milling process force measurements, is discussed in [1]. Kinematic-integrated approaches (setups 2x) and force control using these measuring systems are topics of future work.

**Figure 2:**
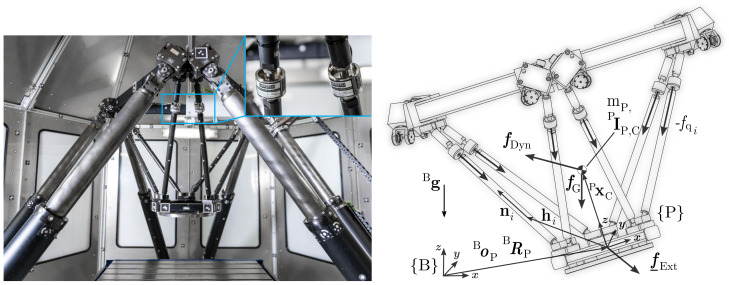
**Left**: Hexapod machine tool with rigid hexapod-based end-effector platform (setup 1b) including six structure-integrated 1 DoF force sensors of the type Althen ALF256 5 kN. **Right**: Corresponding values for measurement model. The integration of the sensors, which are screwed in after cutting, drilling, and thread-cutting the bars of the existing end-effector, is fast, cheap, and does not change mass or size of the end-effector, significantly.

However, every model needs a parametrisation and to make the promising results of [1] available for practical purposes, this paper applies and evaluates parameter identification procedures on the example of the rigid hexapod-based end-effector (setup 1b), Figure 1 and Figure 2. Nevertheless, the introduced methods are also applicable for the remaining structure-integrated setups and even for the use of FT sensors.

For all approaches, the parameters contain sensor positions and orientations (36 parameters, constant for rigid frameworks) and sensor zero offsets (6 parameters). Additionally, for the focused moving rigid bar structures (setup 1b), mass, centre of gravity and inertia of the framework (10 parameters), as well as its actual pose, velocity and acceleration in real-time, are included. Moreover, additional parameters need to be introduced to include influences concerning the sensibility of force sensors towards lateral forces, torques, elastic structure deformation or temperature effects.

Table 1 gives an overview of the presented steps and variations according to the methods and the structure of the approach: Based on the measurement model (Section 3), at first, different identification models are derived, which include rigid body, sensor and error compensation parameters (Section 4). As the description and optimisation of the parameter set are crucial for identification, next, multiple parametrisations are analysed and compared using measurement simulation, Pearson correlation and QR decomposition (Section 5). Hereby, two excitation approaches are presented: a quasi-static measurement at independent poses (Section 6), and a dynamic excitation using continuous trajectories (Section 7). The two excitations are created both, in a manual way and in an algorithmic way by the use of different optimisation criteria and methods. Finally, both methods are compared in terms of the application and identification of reference payloads (Section 8).

## 3. Measurement Model

The measurement model in the current form was first presented in [1] and is summarised here, since it is essential for the identification model. Solving the equations of motion ∂p/∂t=∑f at the platform frame {P}, Figure 2 right, gives external spatial forces and moments ${\underline{f}}_{\mathrm{Ext}}$ that act on the end-effector on the basis of the measured and processed sensor forces $f_{q}^{*}$:

(1) $$\begin{array}{c} {}^{P}{\underline{f}}_{\mathrm{Ext}}={}^{P}{\underline{f}}_{\mathrm{Dyn}}-{}^{P}{\underline{f}}_{G}-{}^{P}J_{P}^{-T}\cdot f_{q}^{\;*}. \end{array}$$

The sensor forces are transformed to Cartesian space using the geometric Jacobian ${}^{P}J_{P}$ of the platform structure, Figure 2 right,

(2) $${}^{P}J_{P}^{-T}=\left(\begin{array}{ccc} n_{1} & \cdots & n_{6}\\ h_{1}\times n_{1} & \cdots & h_{6}\times n_{6} \end{array}\right),$$

and gravity forces ${}^{P}{\underline{f}}_{G}$ are included by

(3) $${}^{P}{\underline{f}}_{G}=m_{P}\left(\begin{array}{c} {}^{B}R_{P}^{T}\;{}^{B}g_{0}\\ {}^{P}x_{C}\times {}^{B}R_{P}^{T}\;{}^{B}g_{0}, \end{array}\right),$$

with the gravitational acceleration ${}^{B}g_{0}$ as well as mass $m_{P}$, orientation ${}^{B}R_{P}$, and centre of gravity ${}^{P}x_{C}$ of the platform. Underlines indicate 6 DoF vectors; upright letters constants. The spatial dynamic force vector ${}^{P}{\underline{f}}_{\mathrm{Dyn}}$ divides in forces ${}^{P}f_{\mathrm{Dyn}}$ and moments ${}^{P}m_{\mathrm{Dyn}}$:

(4) $$\begin{array}{cc} {}^{P}f_{\mathrm{Dyn}}= & \;m_{P}\;{}^{B}R_{P}^{T}\;{}^{B}{\ddot{x}}_{C} \end{array}$$

(5) $$\begin{array}{cc} {}^{P}m_{\mathrm{Dyn}}= & \;{}^{P}I_{P}\;{}^{B}R_{P}^{T}\;{}^{B}{\dot{\omega }}_{P}+\left({}^{B}R_{P}^{T}\;{}^{B}\omega_{P}\right)\times \left({}^{P}I_{P}\;{}^{B}R_{P}^{T}\;{}^{B}\omega_{P}\right)+{}^{P}x_{C}\times {}^{P}f_{\mathrm{Dyn}}, \end{array}$$

with inertia ${}^{P}I_{P}={}^{P}I_{P,C}+m_{P}\;S\left({}^{P}x_{C}\right)\;S\left({}^{P}x_{C}\right)$, angular velocity ${}^{B}\omega_{P}$, and acceleration ${}^{B}{\dot{\omega }}_{P}$ of the platform in relation to the base frame {B}. Here, S(r) is the cross product matrix: S(r)b=r×b. With the platform position ${}^{B}o_{P}$, the centre of gravity acceleration ${}^{B}{\ddot{x}}_{C}$ is

(6) $$\begin{array}{cc} {}^{B}{\ddot{x}}_{C}= & {}^{B}{\ddot{o}}_{P}+{}^{B}{\dot{\omega }}_{P}\times {}^{B}R_{P}{}^{P}x_{C}+{}^{B}\omega_{P}\times \left({}^{B}\omega_{P}\times {}^{B}R_{P}{}^{P}x_{C}\right). \end{array}$$

The platform pose $\underline{x}$ calculates from measured drive positions q in forward kinematics (FK) of the hexapod and the platform velocities $\underline{v}$ from measured drive velocities $\dot{q}$ in differential kinematics with the help of the geometric Jacobian of the hexapod $J_{\mathrm{Hex}}\left(\underline{x}\right)$

(7) $$\begin{array}{cc} \underline{x}= & {\left(\begin{array}{cc} {}^{B}o_{P}^{T} & {}^{B}\phi_{P}^{T} \end{array}\right)}^{T}=FK\left(q\right) \end{array}$$

(8) $$\begin{array}{cc} \underline{v}= & {\left(\begin{array}{cc} {}^{B}{\dot{o}}_{P}^{T} & {}^{B}\omega_{P}^{T} \end{array}\right)}^{T}=J_{\mathrm{Hex}}\left(\underline{x}\right)\;\dot{q}. \end{array}$$

Numeric differentiation of $\dot{q}$ and symbolic differentiation of $J_{\mathrm{Hex}}$ give the acceleration $\dot{\underline{v}}$ of the platform

(9) $$\dot{\underline{v}}={\left(\begin{array}{cc} {}^{B}{\ddot{o}}_{P}^{T} & {}^{B}{\dot{\omega }}_{P}^{T} \end{array}\right)}^{T}=J_{\mathrm{Hex}}\left(\underline{x}\right)\;\ddot{q}+{\dot{J}}_{\mathrm{Hex}}\left(\underline{x},\underline{v}\right)\;\dot{q}.$$

Finally, this leads to the measurement model in its canonical form, with ${}^{P}\omega ={}^{B}R_{P}^{T}\;{}^{B}\omega_{P}$, ${}^{P}v={}^{B}R_{P}^{T}\;v$ etc.,

(10) $$\begin{array}{cc} {}^{P}{\underline{f}}_{\mathrm{Ext}}\left(\underline{x},\underline{v},\dot{\underline{v}}\right)= & \underset{M_{P}}{\underbrace{\left[\begin{array}{cc} m_{P}1 & m_{P}\;S{\left({}^{P}x_{C}\right)}^{T}\\ m_{P}\;S\left({}^{P}x_{C}\right) & {}^{P}I_{P} \end{array}\right]}}\left[\begin{array}{c} {}^{P}\dot{v}\\ {}^{P}\dot{\omega } \end{array}\right]+\underset{C_{P}}{\underbrace{\left[\begin{array}{cc} 0 & m_{P}\;S\left({}^{P}\omega \right)\;S{\left({}^{P}x_{C}\right)}^{T}\\ 0 & S\left({}^{P}\omega \right)\;{}^{P}I_{P} \end{array}\right]}}\left[\begin{array}{c} {}^{P}v\\ {}^{P}\omega \end{array}\right]\\ + & \underset{{\underline{g}}_{P}}{\underbrace{\left[\begin{array}{c} -m_{P}{}^{B}R_{P}^{T}\;{}^{B}g_{0}\\ -m_{P}\;S\left({}^{P}x_{C}\right){}^{B}R_{P}^{T}\;{}^{B}g_{0} \end{array}\right]}}-{}^{P}J_{P}^{-T}f_{q}^{\;*}\\ = & M_{P}\;{}^{P}\dot{\underline{v}}+C_{P}\;{}^{P}\underline{v}+{\underline{g}}_{P}-{}^{P}J_{P}^{-T}f_{q}^{\;*}. \end{array}$$

Here, $f_{q}^{\;*}$ stands for compensated sensor values that already contain corrections and calculates to

(11) $$f_{q}^{\;*}=f_{q}-f_{q0}-f_{K}\left(\underline{x}\right).$$

For the present setup, these are sensor zero offsets ${\underline{f}}_{q0}$ and pose-dependent influences ${\underline{f}}_{K}\left(\underline{x}\right)$, which address lateral sensor forces and moments from platform deformation, as described next.

According to the rigid body assumption of the end-effector, the force measurement is expected to be independent of the platform position, Equation (10). However, experiments show a not negligible position-dependent force deviations in a range of ±20N (within a measuring range of ≈9 kN in XY) that are strictly systematic and mainly dependent on X- and Y-coordinates [1]. Ref. [2] evaluates the platform deformation that results from the 36 varying platform joint forces and moments, which are applied by the hexapod kinematic struts, as reasonable explanation for this behaviour through an analysis based on a finite element model (FEM). As a full elastic model has disadvantages in parametrisation and online calculation in the control system, the effect is compensated by a prediction model with the choice of

(12) $$f_{K1}=K_{1}\;{\left({}^{B}x_{P}\;\;\;{}^{B}y_{P}\right)}^{T},$$

where the hexapod pose is used to describe the current static load situation of platform forces. This approach produces two linear factors for each sensor, that can be identified by quasi-static measurements. Evaluations show that these 12 parameters do not represent a minimal model. Transforming $K_{1}$ to Cartesian, reveals a systematic force deviation that can be described in a reduced form as a second modelling approach

(13) $$f_{K2}={}^{P}J_{P}^{T}\left(\begin{array}{c} k_{0}\;1\\ S\left({\left(\begin{array}{ccc} 0 & 0 & k_{1} \end{array}\right)}^{T}\right) \end{array}\right)\;\left(\begin{array}{c} {}^{B}x_{P}\\ {}^{B}y_{P}\\ 0 \end{array}\right)={}^{P}J_{P}^{T}\;K_{2}\left(\begin{array}{c} {}^{B}x_{P}\\ {}^{B}y_{P}\\ 0 \end{array}\right).$$

Now, the effect is compensated for by only two parameters $\left\{k_{0},k_{1}\right\}$.

In fact, platform deformations resulting from platform joint forces not only occur at position changes but also at orientation changes. Neglecting these influences leads to the incorrect identification of mass and centre of gravity by quasi-static measurements, as shown below. As the orientation is part of the rigid body gravity model, Equation (3), a compensation approach will correlate with the parameters mass $m_{P}$ and centre of gravity ${}^{P}x_{C}$. Therefore, identification is not possible when the force offsets are modelled in any formulation using the orientation matrix, such as $f_{K3}=f_{K1}+K_{3}\;{}^{B}R_{P}^{T}\;{}^{B}g$. To separate the parameters, a different angle format can be used for the orientation error model, Equation (14), as an extension to Equation (12)

(14) $$f_{K4}=f_{K1}+K_{4}\;\phi .$$

Again, a transformation to Cartesian shows that the parameters can be reduced to the two most significant influences $k_{2}$ and $k_{3}$

(15) $$f_{K5}=f_{K2}+{}^{P}J_{P}^{T}\left(\begin{array}{c} S\left({\left(\begin{array}{ccc} 0 & 0 & k_{2} \end{array}\right)}^{T}\right)\\ \left(\begin{array}{ccc} k_{3} & k_{3} & 0 \end{array}\right)\;1 \end{array}\right)\phi ={}^{P}J_{P}^{T}\;K_{5}\;\phi .$$

For this notation, an axis based format—here the Gibbs vector—needs to be used. A change of the Jacobian due to platform deformation can be neglected.

In conclusion, by those four parameters, $\left\{k_{0},k_{1},k_{2},k_{3}\right\}$, the pose-dependent force deviations are compensated for by more than factor 10 to a remaining total error of approximately ±2N (3 s, 99.73 %) in XY for the measurement at quasi-static poses in the workspace [1].

Alternatively, by inserting joints [12,15] or flexure hinges [13,25] into the structure, forces lateral to the sensors can be reduced mechanically. Nevertheless, they cannot remove longitudinal forces that result from a deformation of the fundamental frame, which here is the platform part that deforms due to forces applied by the hexapod struts. Further, joints and flexure hinges introduce backlash or reduce the platform stiffness respectively, which is not desired in machine tools.

## 4. Identification Model

Besides process force measurement and control, structure-integrated force sensors can be used for parameter identification: As mass, centre of gravity and inertia of the end-effector can change during machine usage, for example through a workpiece or tool change, a fast and easy procedure for identifying these parameters is required, which should last less than 5min and can run in a production break. Other parameters, such as sensor offsets and error compensation factors, are only affected marginally by load changes but need to be determined at least once after system assembly. Generally, it is advantageous to identify all parameters at the same time to achieve the least global variance. If the system has been identified, a pure sensor drift can be tared without further measurements by adjusting only $f_{q0}$, Equation (11).

Since friction and stick-slip effects can be neglected for the sensor placement in the rigid end-effector without joints (setup 1b), an identification from quasi-static measurements is possible: Doing this, the 10 parameters, platform mass $m_{P}$, centre of gravity ${}^{P}x_{C}$ and force sensor offsets $f_{q0}$, as well as the pose-dependent compensation offsets K can be identified. This procedure is appropriate for quasi-static or continuous applications that do not require inertia, which cannot be identified statically. However, limited dynamics apply for most applications in force measurement and control to reduce measuring noise. On the other hand, a measurement during accelerated machine movement allows a parameter identification from dynamic excitations and, additionally, the identification of the platform inertia ${}^{P}I_{P},C$. With this, both standard approaches to identification are discussed: the quasi-static identification from independent poses and the identification from dynamic excitation trajectories with dependent data points. The Jacobian, Equation (2), is not identified since it is, as a pure geometrical transformation, already known from mechanical drawings. In the rare case when it is unknown, it can be determined directly by geometric measurements far more accurately than indirectly from dynamic identification.

Below, a linear identification model of the form

(16) $$f_{q}=A\;u$$

is introduced for the complete system. A partial identification model, for example, without inertia, can be achieved by elimination of the corresponding columns in the regressor matrix A and rows in u. First, the vector u of u unknowns is set to

(17) $$u={\left(\begin{array}{ccccc} m_{P} & m_{P}{}^{P}x_{C}^{T} & {}^{P}{\bar{I}}_{P}^{T} & f_{q0}^{T} & {\bar{K}}^{T} \end{array}\right)}^{T},$$

where matrices have been transformed to vectors, which is indicated by an overline: ${}^{P}{\bar{I}}_{P}=\left\{I_{11},I_{12},I_{13},I_{22},I_{23},I_{33}\right\}$. Rearranging the model from Equation (1) to express n virtual sensor forces ${\hat{f}}_{q}$ gives the non-linear identification model

(18) $$\begin{array}{c} {\hat{f}}_{q}\left(\underline{x},\underline{v},\dot{\underline{v}},u\right)={}^{P}J_{P}^{T}\left[{}^{P}{\underline{f}}_{\mathrm{Dyn}}-{}^{P}{\underline{f}}_{G}\right]+f_{q}0+f_{K}. \end{array}$$

The identification procedure can be performed with the non-linear model, Equation (18), by calculating its local Jacobian and iterating over it or by transforming Equation (18) into a linear form analytically, which is advantageous, as no iterations are needed to solve the problem. The linear form of the rigid body part can directly be obtained from Equation (10)

(19) $$\begin{array}{cc} A_{B} & (\underline{x},\underline{v},\dot{\underline{v}})=\\ \; & \left(\begin{array}{ccc} \dot{v}-g & S\left(\dot{\omega }\right)+S\left(\omega \right)S\left(\omega \right) & 0\\ 0 & S{\left(\dot{v}-g\right)}^{T} & L\left(\dot{\omega }\right)+S\left(\omega \right)L\left(\omega \right) \end{array}\right), \end{array}$$

with the help of the operator L(ω) [22,24]

(20) $$I\omega =L\left(\omega \right)\;\bar{I}=\left(\begin{array}{cccccc} \omega_{1} & \omega_{2} & \omega_{3} & 0 & 0 & 0\\ 0 & \omega_{1} & 0 & \omega_{2} & \omega_{3} & 0\\ 0 & 0 & \omega_{1} & 0 & \omega_{2} & \omega_{3} \end{array}\right)\bar{I}.$$

Together with the measurement parameters, this gives the linear form for one dataset j=1…m

(21) $${\hat{f}}_{qj}=A_{j}\left({\underline{x}}_{j},{\underline{v}}_{j},{\dot{\underline{v}}}_{j}\right)\;u=\left(\begin{array}{ccc} {}^{P}J_{P}^{T}\;A_{Bj} & 1 & \tilde{K}\left({\underline{x}}_{j}\right), \end{array}\right)u.$$

where 1 is the identity matrix and $\tilde{K}$ represents compensation factors of $f_{K}$. In the case of Equation (12) $\tilde{K}$ is ${\tilde{K}}_{1}=\tilde{L}\left({\underline{x}}_{j}\right)$, where $\tilde{L}$ provides a transformation $K_{1}\;\underline{x}=\tilde{L}\left(\underline{x}\right)\;{\bar{K}}_{1}$ similar to the inertia linearisation. In the case of Equation (13) $\tilde{K}$ is

(22) $${\tilde{K}}_{2}={}^{P}J_{P}^{T}\left(\begin{array}{cc} {\left({}^{B}x_{Pj}\;\;\;{}^{B}y_{Pj}\;\;\;0\right)}^{T}\; & 0\\ 0 & {\left({}^{B}y_{Pj}\;\;\;-{}^{B}x_{Pj}\;\;\;0\right)}^{T} \end{array}\right),$$

with the unknowns ${\bar{K}}_{2}=\left\{k_{0},\;k_{1}\right\}$, and for $K_{3}$, $K_{4}$, and $K_{5}$ in a similar way.

Stacking m measurements ${\overset{ˇ}{f}}_{qj}$ and the corresponding models $A_{j}$ to the overdetermined global system leads to

(23) $${\overset{ˇ}{f}}_{q}=Au+\varepsilon ,$$

which can be solved by the use of a Gauss-Markov approach

(24) $$\hat{u}={\left[A^{T}\;W\;A\right]}^{-1}A^{T}\;W\;{\overset{ˇ}{f}}_{q},$$

with the weighting matrix $W=\mathrm{cov}{\left(f_{q}\right)}^{-1}$ [26]. The weighting matrix can be used to take varying characteristics of the measurements into account, regarding different units or the individual measuring accuracy of different sensors. As only forces are measured and identical sensors are used in the present setup (ALF256 5 kN, Althen GmbH Mess- und Sensortechnik, Kelkheim, Germany), task variable scaling is not necessary and W is set to identity, which reduces the problem to an ordinary least-squares regression.

In contrast, parameter scaling is still required to obtain meaningful and comparable singular values for the next steps model analysis and optimisation as well as improved convergence. With the scaling matrix $H=\mathrm{diag}(h_{1}\ldots h_{u})$, the model changes to [24]

(25) $${\overset{ˇ}{f}}_{q}=\left(AH\right)\left(H^{-1}u\right)=\tilde{A}\tilde{u}+\varepsilon .$$

For the choice of H, column scaling is used, as it is simple and does not require a priori statistical data, with $a_{l}$ as *l*th column of A [27]

(26) $$h_{l}=\left\{\begin{array}{cc} \left|\right|a_{l}{\left|\right|}^{-1} & \left|\right|a_{l}\left|\right|\neq 0\\ 1 & \left|\right|a_{l}\left|\right|=0. \end{array}\right.$$

An estimate of the global standard deviation of the regression problem can be obtained from the $\chi^{2}$ statistic [24,26]

(27) $${\hat{s}}_{0}=\sqrt{\frac{\chi^{2}}{m\cdot n-u}},\;\mathrm{with}\;\chi^{2}={\left({\overset{ˇ}{f}}_{q}-A\hat{u}\right)}^{T}W\left({\overset{ˇ}{f}}_{q}-A\hat{u}\right),$$

the number of poses m, the number of forces measured per pose n, and the number of unknowns u. The covariance matrix as well as local standard deviations of the unknowns give

(28) $$\hat{M}={\hat{s}}_{0}^{2}\;F^{-1}\;\mathrm{and}\;{\hat{s}}_{j}=\sqrt{{\hat{M}}_{jj}}.$$

## 5. Model and Identifiability Analysis

Generally, only those parameters that are modelled are identifiable, stimulated by the exciting trajectory (sensitivity), and that can be distinguished from each other (linear independence). Therefore, the optimisation of the parameter set and the optimisation of the exciting data points or trajectory parameters, respectively, are the two major steps in identification preparation. The parameters can be grouped in identifiable and unidentifiable parameters as well as parameters that are only identifiable in linear combination, expressed by a new parameter. QR or singular value decomposition of $\tilde{A}$ helps in analysing the identifiability of the parameter set [24].

For the end-effector, different models are proposed for identification with different parameter sets and properties (Table 2). Identification results for these models are shown in Table 3. Generally, the inertia ${}^{P}I_{P}$ is not identifiable by quasi-static measurements. Model 0 contains the basic static rigid body parameters and sensor offsets but does not include error correction factors. Model 1, Equation (12), and model 2, Equation (13), include position-dependent error correction, which increases the measurement accuracy significantly [1], where model 2 describes this effect with fewer parameters, as described above.

For all static models that neglect a modelling of orientation-dependent force errors (models 0–2), the parameters $m_{P}^{*}$ and ${}^{P}x_{C}^{*}$ do not represent the physical platform mass and centre of gravity, which results in an empirical model rather than in a physical model. Still, the models are valid because these parameters include the mentioned effects. Changes of mass and centre of gravity, on the other hand, can be identified with high precision and the correct physical values [2]. This is important, as tool or workpiece parameters are represented with physical meaning.

**Figure 3:**
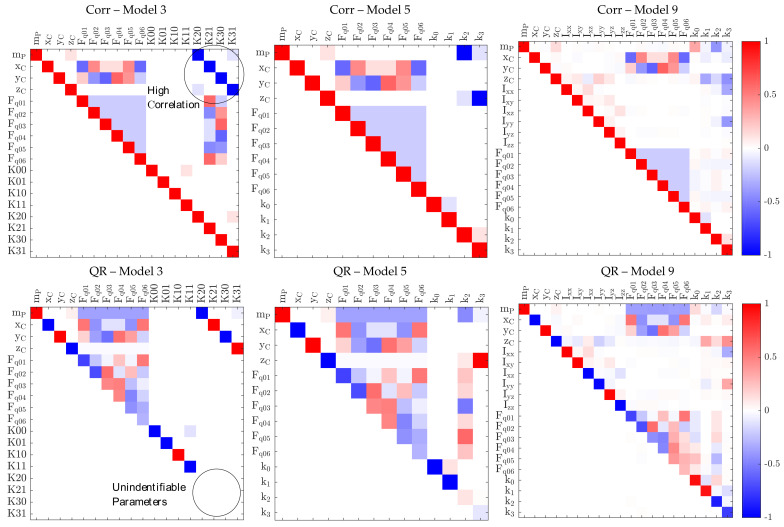
Matrix of Pearson correlation coefficients (Corr) and upper R-part of QR decomposition of $\tilde{A}$ (QR); **left**: model 3 (not identifable in any description based on $R^{T}g$), **centre**: preferred quasi-static model 5 (includes models 0, 2), **right**: preferred dynamic model 9 (includes models 6, 7). The models 1, 4, 8 are not displayed due to too many parameters. Corr shows the pairwise linear correlation between 2 parameters, that points out linear dependencies. In R high values in the row behind the diagonal element indicate dependent parameters. The diagonal elements $R_{ii}$ of R that are less than the numerical zero $\left(m\cdot n\cdot \epsilon \cdot \max|R_{ii}|\right)$ indicate unidentifiable parameters $u_{i}$ [24].

Including orientation-dependent force errors in the models always leads to a correlation of the error model parameters to the rigid body parameters, mass and centre of gravity, for quasi-static measurements since both parameter sets can only be excited by inclinations. When modelled by rotation matrix, Equation (15), the parameters are not identifiable as the mathematical representation is too close to the rigid body gravity model in Equation (3). Figure 3 left shows this effect in plots of correlation and QR decomposition of the scaled regressor matrix $\tilde{A}$ for model 3.

Including orientation-dependent force errors, based on a different orientation representation, leads to the model 4, Equation (14), and model 5, Equation (15), which allows for the identification of mass and centre of gravity with correct physical meaning, where model 5 describes the effect with less parameters.Still, the correlation between these parameters is a physical fact that cannot be completely overcome by a different modelling for quasi-static measurements so that the uncertainties stay high, as can be seen in Figure 3 centre.

Even though models 0–2 are valid and contain fewer parameters, a structural rigid body approach using parameters with physical meaning is advantageous for a second reason: as Table 2 shows, dynamic identification results in correct rigid body parameters for models 6 and 7 without orientation-dependent force error models. This is because a dynamic trajectory excites the parameters in a different way, while the platform joint forces that cause the force errors through platform deformation are foremost inducted by static hexapod strut forces—dynamic joint forces are much smaller and can be neglected, as separate simulations show. For the same reason, model 3 becomes identifiable for dynamic excitations when expanded by an inertia model (not displayed).

Finally, models 8 and 9 include all error compensation parameters for dynamic identification, where model 9 uses fewer parameters (Figure 3 right).

For a symmetric platform without accessories, some centre of gravity parameters $\left\{{}^{P}x_{\mathrm{Cx}},{}^{P}x_{\mathrm{Cy}}\right\}$ and moments of deviation $\left\{{}^{P}I_{\mathrm{xy}},{}^{P}I_{\mathrm{xz}},{}^{P}I_{\mathrm{yz}}\right\}$ may also be eliminated, which reduces the model by 5 more parameters. On the other hand, in practice, a workpiece can be applied at any position at the end-effector making these parameters necessary, which is why this reduction is not performed in the present case.

In conclusion, models 5 and 9 provide a full error correction with minimal parameters and are chosen as the basis for further steps in the evaluation of quasi-static and dynamic identification procedures, respectively.

In order to optimise poses and exciting trajectories, criteria are needed that can be defined on the basis of the regression matrix A and on the inverse Fisher information matrix

(29) $$F^{-1}\left(x\right)={\left(A^{T}\left(x\right)\;W\;A\left(x\right)\right)}^{-1},$$

as presented in Table 4.

**Table 4:** Observability indicies and optimization criteria [24,28].

Criteria	Expression	Description
A	(30) $\mathrm{trace}(F^{-1}\left(x\right))\to \min$	Minimize sum of unknown variances (average variance).
D	(31) det(F(x))→max	Maximize volume of parameter uncertainty hyperellipsoid.
K	(32) $\kappa \left(A\left(x\right)\right)=\mu_{1}/\mu_{u}\to \min$	Minimize condition as eccentricity of the hyperellipsoid.
N	(33) $\mu_{u}^{2}/\mu_{1}\to \max$	Maximize *noise amplification index*.

## 6. Quasi-Static Identification Measurements

For quasi-static identification measurements, several poses are approached in the workspace and, at each pose, a set of force samples is taken in a stationary condition. The ability to wait until dynamic effects extinguish, and to average over many samples, leads to a higher data point quality for quasi-static measurements when compared to dynamic measurements. At the same time, the data points are independent so that every data point is valid; the data do not need to be cut and, generally, fewer data points are acquired, which is advantageous concerning memory and processing time of the regression. Still, the measuring time is very high and the inertia cannot be identified.

Depending on the model, a minimum of m = u/n poses is required, where an overdetermined measurement including approximately 10 times more poses than necessary aims to obtain regression results with acceptable uncertainty. The choice of the correct poses is crucial for good identification results, where different parameters are excited by different poses: The identification of $\{m_{P}$, ${}^{P}x_{C}\}$ and $\left\{k_{2},k_{3}\right\}$ requires large inclinations to excite varying gravity forces in Equation (3) and platform joint forces compensated by Equation (15), respectively. The identification of $\{k_{0}$, $k_{1}\}$, Equation (13), requires large position changes in XY directions.

Optimising the pose set by selecting the most sensitive poses avoids redundancies and, in total, reduces both poses and measuring time, significantly. Basically, two approaches are available to optimise the dataset: As the data points are independent, poses can be added randomly until the chosen criterion saturates. Alternatively, starting from a generated good dataset, poses can be removed as long as the chosen criterion does not change significantly. Here, the latter approach is performed using the criteria shown in Table 4. For every pose set, one pose is deleted temporarily and the criterion is calculated. After examining all poses, the pose with the smallest change of the selected criterion is found as pose with least influence and deleted permanently. Finally, this procedure repeats until the algorithm reaches the desired number of poses (Algorithm 1).

**Figure 4:**
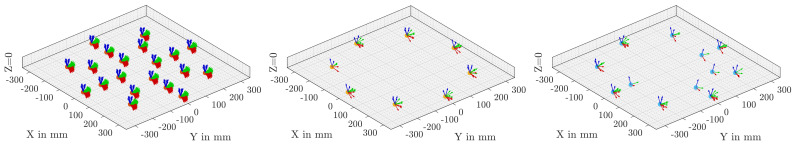
Poses for quasi-static parameter identification in the Cartesian workspace, **left**: original dataset with 480 poses (Q5M); **centre** and **right**: reduced datasets (30 poses each) after optimization with A (Q5A) and N (Q5N) criteria, respectively.

Table 5 shows results for a reduction to 30 poses starting from a working dataset of 480 generated poses that include position changes from −300 mm to 300 mm in steps of 100 mm in XY and, at each position, orientation changes from 10 ° to 20 °, 0 ° to 270 ° and −15 ° to 15 ° in steps of 10 °, 90 ° and 15 °, respectively, for modified Euler angles [29], Figure 4 left.


**Algorithm 1:** Selection of optimal poses using criteria of Table 5.  // Create a set of valid poses  **for**
$x\leftarrow x_{\min}$
**to**
$x_{\max}$
**by**
$x_{\mathrm{step}}$
**do**   **for**
$y\leftarrow y_{\min}$
**to**
$y_{\max}$
**by**
$y_{\mathrm{step}}$
**do**    **for**
$z\leftarrow z_{\min}$
**to**
$z_{\max}$
**by**
$z_{\mathrm{step}}$
**do**     **for**
$a\leftarrow a_{\min}$
**to**
$a_{\max}$
**by**
$a_{\mathrm{step}}$
**do**      **for**
$b\leftarrow b_{\min}$
**to**
$b_{\max}$
**by**
$b_{\mathrm{step}}$
**do**       **for**
$c\leftarrow c_{\min}$
**to**
$c_{\max}$
**by**
$c_{\mathrm{step}}$
**do**        **if**
*is*_*valid*(*pose*(*x*,*y*,*z*,*a*,*b*,*c*)) **then**         pose_list.add(pose(x,y,z,a,b,c));  // Reduce from n to r optimal poses
  red_pose_list←pose_list;  **for**
i←1
**to**
n−r
**do**
   crit0←calc_criteria(red_pose_list);   **for**
j←1
**to**
length(red_pose_list)
**do**    test_list←red_pose_list;    test_list.remove_pose(j);    crit(j)←calc_criteria(test_list);   red_pose_list.remove(index_of(min(crit0−crit)));


This optimisation reduces the measuring time from 21 min for 480 poses to 81 s for 30 poses (with approximately 2.6 and 2.7 s per pose, respectively), which meets the requirement of a fast measurement in production breaks and, at the same time, realises an over-determination factor of 12.8. Further, the algorithm can be run until A loses rank, which gives the minimum number of possible poses and, therefore, proves the selection of good poses through the algorithm, as the number is close to or reaches the theoretical minimum of 3 poses for all criteria (Table 5). A reduction to less than 30 poses is possible but not recommended since it increases the parameter uncertainties.

All criteria reduce the data set to 30 valid poses. As expected, poses with high absolute position values and large inclinations are preferred, whereas centred poses and poses with small inclinations are neglected (Figure 4 centre and right). For all parameters and criteria, the standard deviations increase by approximately factor 5. The most sensitive parameters regarding the data reduction are $\left\{k_{2},k_{3}\right\}$ and $\{m_{P}$, ${}^{P}x_{C}\}$, as the standard deviations are already higher without reduction. This matches the expectations from the correlation and QR plots in Figure 3, which indicate this correlation. Even when the condition criteria K shows the smallest total standard deviation ${\hat{s}}_{0}$ of the optimised solutions, the plots reveal a displacement of the averages for several parameters when compared to the not-optimised solution. However, all optimised solutions are valid and do not differ very much. Besides validating the approach, this indicates that in the presented case, the previous step—meaning the model optimisation—is far more important than the used criteria for pose optimisation.

## 7. Dynamic Identification Measurements

Dynamic parameter identification measurement stands for the online acquisition of data points during a highly accelerated movement of the end-effector. Using a dynamic excitation trajectory enables the collection of many data points in a short time and the identification of the platform inertia that cannot be identified by quasi-static measurements. On the other hand, if an individual data point of the trajectory has a lower quality, a pre-processing of the data may be necessary and the regression needs more calculation time owing to more data points when compared to quasi-static measurements. Filtering options are also limited, the reconstruction of the necessary acceleration can be challenging and a synchronous measurement of force and acceleration needs to be ensured beforehand. With the use of 16 Bit analogue inputs EL3742 and EL3702 (Beckhoff, Verl, Germany), which are connected to a local EtherCAT-Client EK1101 (Beckhoff, Verl, Germany) at the end-effector platform, a fast and synchronous data acquisition of the forces is realised. The oversampling functionality allows a data acquisition rate up to 100 kSamples/s (10 s) that is used for 100 times averaging during a task cycle of 1 ms. Further, strut positions and velocities are acquired synchronously from a Sercos II drive bus, where velocities are differentiated to accelerations numerically. Finally, filter times of force and acceleration are adjusted to obtain a synchronous data acquisition, where the differentiator delay needs to be taken into account. Details of data acquisition and measurement results, for example, during a milling process, are presented in [1].

For the exciting trajectory, two approaches are presented: a manually created trajectory and an optimised trajectory based on periodic functions. The model is so simple that the individual parameter sensitivity with regard to specific movements can be estimated by examining the equations, compare Equation (10), Equation (13) and Equation (15). A trajectory that is sufficient for parameter identification can be found considering the following aspects: the parameters mass $m_{P}$ and centre of gravity ${}^{P}x_{C}$ are excited best by accelerations, which can be created by gravitational acceleration using inclinations (${}^{B}R_{P}^{T}\;{}^{B}g_{0})$, as in the quasi-static approach, or by highly accelerated dynamic movements in translational directions ${}^{P}\dot{v}$, where the latter is advantageous as it reaches higher magnitudes and avoids correlation to the error correction factors $\left\{k_{2},k_{3}\right\}$. The inertia requires high angular accelerations ${}^{P}\dot{\omega }$ and the error correction factors $\{k_{0}$, $k_{1}\}$ require large position changes with low acceleration.

**Figure 5:**
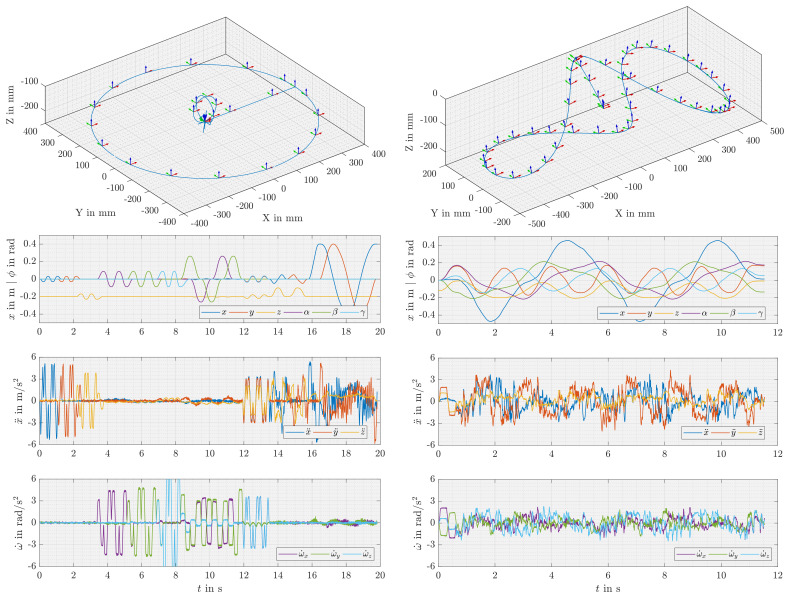
Trajectories and corresponding measured accelerations for dynamic parameter identification; **left**: manually designed excitation trajectory with independent excitation of the Cartesian directions (D9M), **right**: excitation trajectory resulting from N-optimal 3rd order periodic Fourier series (D9N).

This leads to a trajectory, as shown in Figure 5 left, that contains successive excitations in all Cartesian directions {x,y,z,α,β,γ} followed by slow inclinations, a fast 6 DoF motion, and 3 circles placed in the 3 Cartesian planes, where the latter has a large diameter to excite $\{k_{0}$, $k_{1}\}$. The measured accelerations show clear excitations in the respective directions, except for slower or more complex movements, where they become noisy.

Optimising a trajectory, on the other hand, requires criteria that are defined in Table 4 and, as the data points are not independent, a proper description of the trajectory that is smooth, closed and limited with respect to the workspace borders. Periodic Fourier series, as presented by [30] for serial kinematics and applied for parallel kinematics by [20], meet these requirements and are used here as well.

For every DoF i=1…6 in the workspace, a movement is defined as follows: [20,30]

(34) $$\begin{array}{cc} {}^{i}x\left(t\right) & =\sum_{l=1}^{N}\left(\frac{{}^{i}a_{l}}{l\omega }\sin\left(l\omega t\right)-\frac{{}^{i}b_{l}}{l\omega }\cos\left(l\omega t\right)\right)+{}^{i}x_{0} \end{array}$$

(35) $$\begin{array}{cc} {}^{i}\dot{x}\left(t\right) & =\sum_{l=1}^{N}\left({}^{i}a_{l}\cos\left(l\omega t\right)+{}^{i}b_{l}\sin\left(l\omega t\right)\right) \end{array}$$

(36) $$\begin{array}{cc} {}^{i}\ddot{x}\left(t\right) & =\sum_{l=1}^{N}\left(-{}^{i}a_{l}l\omega \sin\left(l\omega t\right)+{}^{i}b_{l}l\omega \cos\left(l\omega t\right)\right). \end{array}$$

The analytical availability of the derivatives is advantageous, as they can be used to filter the usually noisy velocity or acceleration measurements. Depending on the angle format, $\underline{v}$ and $\dot{\underline{v}}$ can be obtained from $\dot{\underline{x}}$ and $\ddot{\underline{x}}$ by the well known transformation

(37) $$\begin{array}{cc} \underline{v} & =T\dot{\underline{x}} \end{array}$$

(38) $$\begin{array}{cc} \dot{\underline{v}} & =T\ddot{\underline{x}}+\dot{T}\dot{\underline{x}},\mathrm{with} \end{array}$$

(39) $$\begin{array}{cc} T & =\left(\begin{array}{cc} 1 & 0\\ 0 & R(\Phi ,\Theta ) \end{array}\right)\;\mathrm{and}\;R=\left(\begin{array}{ccc} c_{\Theta }c_{\Psi } & -s_{\Psi } & 0\\ c_{\Theta }s_{\Psi } & c_{\Psi } & 0\\ -s_{\Theta } & 0 & 1 \end{array}\right), \end{array}$$

on the example of Roll-Pitch-Yaw angles {Φ,Θ,Ψ}.

Further, the trajectory must be kept within valid bounds during optimisation. Basically, the hexapod movement is limited by strut lengths, velocities and accelerations, passive joint angles and singularities. Owing to the specific configuration of the hexapod (e.g., eccentric joints), limited workspace coordinates are sufficient to avoid critical joint angles and singularities with certainty for the present kinematic, as long as they are not chosen very close to the workspace border. Velocity and acceleration limits can also be expressed in workspace coordinates

(40) $$\begin{array}{cc} {\underline{x}}_{\min} & \leq \underline{x}\left(t\right)\leq {\underline{x}}_{\max} \end{array}$$

(41) $$\begin{array}{cc} {\dot{\underline{x}}}_{\min} & \leq \dot{\underline{x}}\left(t\right)\leq {\dot{\underline{x}}}_{\max} \end{array}$$

(42) $$\begin{array}{cc} {\ddot{\underline{x}}}_{\min} & \leq \ddot{\underline{x}}\left(t\right)\leq {\ddot{\underline{x}}}_{\max}. \end{array}$$

Altogether, this approach significantly improves optimisation speed as it avoids the calculation of the inverse kinematic, differential kinematic and validation algorithms inside the optimisation problem. At the same time, the remaining workspace stays large enough to house an optimised trajectory.

Looking at the parameters, the trajectory consists of 6(2N+1)+1 variables ${}^{i}x_{0}$, ${}^{i}a_{l}$, ${}^{i}b_{l}$ and ω that can be optimised, where *N* is the order of the Fourier series and needs to be chosen beforehand. As the trajectory can be centred in the workspace due to machine symmetry, the parameters ${}^{i}x_{0}$ are set to zero, which reduces the problem by 6 parameters. Generally, fewer parameters is advantageous for the convergence of the optimisation algorithm, where low order Fourier series produce very simple trajectories, which make an optimisation pointless, for example, circles for order 1. On the other hand, high order trajectories may easily converge to a local minimum and, further, may contain many direction changes that can break the acceleration constraints or require a reduction of the total velocity, which also reduces the excitation effect of the trajectory. For these reasons, the order should be at least 3 (37 parameters) and not higher than 7 (85 parameters). The presence of very small parameter values can indicate that the order can be reduced. Optimisation calculations, performed using model 9 for the criteria (A, D, K, N) of Table 4, different orders (1–7) and different solvers (‘interior-point’, ‘active-set’ and ‘sqp’ of Matlab R2019a (The Mathworks Inc., Natick, MA, USA) *fmincon* optimiser for constrained non-linear minimisation), show the best results using the ‘active-set’ solver, D- or N-optimal criterion and third to fifth order trajectories, such as those presented in Figure 5 right. Even with these optimisations, which are performed with equal start values, can result in similar trajectories for different solvers and criteria, there are differences in performance and quality. In detail, the ‘active-set’ converges in most cases and needs fewer iterations than ‘sqp’, which sometimes does not converge—‘interior-point’ does not converge in general. Regarding the criteria, the D-optimal criterion, which maximises the volume of the parameter uncertainty hyper-ellipsoid, converges best due to least iterations and uncertainties, as also predicted by other authors for this identification task [17,20]. Further, the N-optimal criterion, which can be seen as a combination of minimising the condition number and maximising the minimum singular value [24,28], performs quite well, as it produces valid excitation trajectories within few iterations and with good parameter identifiability. Finally, A- and K-criterion can also lead to valid trajectories but usually need more iterations and contain weak parameters indicated by QR-analysis and, therefore, are not recommended. Furthermore, the algorithm requires good initial parameters, as it may converge to local minima, which is indicated by different trajectory results on randomly generated start values. On the other hand, even local minima results lead to trajectories that are sufficient for parameter identification. For this reason, random initial values are acceptable when they significantly differ from zero and are within the trajectory bounds. Consequently, the optimisation algorithm using the mentioned settings leads to a fast convergence (mainly depending on solver and criteria), a limited trajectory with significant acceleration (mainly depending on start values and criteria) and significant trajectory parameters (mainly depending on order). In contrast to the quasi-static approach, both optimising the model and choosing the algorithm and criteria are essential for good results.

When comparing the measured accelerations of both trajectories (Figure 5), the manually planned trajectory shows higher magnitudes and far less noise. An explanation for this behaviour can be assumed in two characteristics of the optimised path approach. First, the optimised trajectory is planned as a continuous spatial path in all DoF and is composed of many intermediate points, whereas the manually created trajectory behaves more like a point-to-point motion that realises the successive Cartesian excitations and contains fewer intermediate points. This is significant because the path planner and interpolator in the numeric control (CNC) pre-process the path under the condition of minimal contour deviations including a velocity adjustment during the look-ahead procedure and jerk limitation depending on contour artefacts, such as non tangential block transitions. By activating spline interpolation, allowing contour deviations and improving dynamic parametrisation, a significantly higher dynamic is achieved. Nevertheless, to find a good parametrisation of the CNC path planner for the optimised trajectories containing simultaneous translations and rotations in 6 DoF can be challenging for parallel kinematics. For point-to-point movements instead, the path can be planned with maximal acceleration in all DoF neglecting contour deviations. Second, by the use of 7 segment motion profiles for the point-to-point movements, higher accelerations can be reached in general, when compared to the sin/cos movements of the optimised Fourier series.

Table 6 compares identification results for both dynamic trajectories. Both identify similar rigid body parameters and sensor offsets, where the manually chosen trajectory shows smaller uncertainties in both, ${\hat{s}}_{0}$ and ${\hat{s}}_{j}$, when compared to the periodic trajectory. Further data processing, such as the selection of highly accelerated motion sections or the filtering of the accelerations based on discrete Fourier transformation (DFT-filtering), does not improve the results for the presented application. As a consequence of less measuring noise in accelerations and smaller uncertainties during identification, the manually chosen trajectory is preferred.

## 8. Validation and Comparison

**Figure 6:**
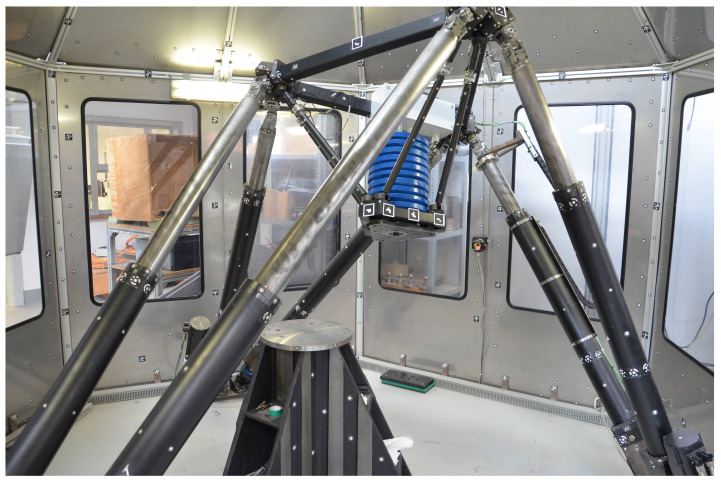
Validation of the approach through identification of known payloads.

As a final step, the identification procedures and exciting poses/trajectories are validated and compared by the application and identification of benchmark payloads (Figure 6). In detail, weights with a total mass of 100 kg are applied to the end-effector in steps of approximately 20 kg and parameters are identified using the presented methods of quasi-static identification (model 5, with all poses, Q5M, and with 30 N-optimal poses, Q5N) and dynamic identification (model 9, manually designed, D9M, and optimised periodic trajectory, D9N).

Table 7 shows the identification results for selected parameters of all measurements as well as relative deviations to the theoretical physical values that result from the application of the known benchmark payloads. For all approaches, the identified masses, centre of gravities and inertias properly change with the applied load and show a relative error that is in large part significantly smaller than the maximum of 3.9%, measured for ${}^{P}z_{C}$ at 100 kg with the Q5N method. Further, the constant parameters $f_{q0}$ remain constant with a maximal deviation of 2.5% ($f_{q02}$ at 100 kg Q5N). The relative errors increase with the applied load, where the average relative error per step is nearly constant, for example, $\Delta {\bar{m}}_{P}=-0.5\;\%/20\;\mathrm{kg}$ for Q5M. The quasi-static methods show a slightly lower accuracy in the identification of the centre of gravity when compared to dynamic methods, as already expected from model analysis in Section 5. Least deviations in mass (<2%) and sensor offsets (<1.1%) shows D9N, where the other methods show slightly higher deviations that are similar between them.

For the dynamic trajectories, the global standard deviation ${\hat{s}}_{0}$ increases significantly with the load (factor 5.4 and 5.8 between 0 kg and 100 kg for D9M and D9N, resp.), whereas the quasi-static method is not affected that much (factor 1.8, Q5M) by the applied loads, even after reducing the number of poses (factor 1.7, Q5N). A reduction of eigenfrequencies with higher applied mass may increase the signal noise during movement and cause this effect. Again, the periodic trajectory D9N leads to higher uncertainties in identification, for example, ${\hat{s}}_{0}=46.6$ at 100 kg, caused by higher acceleration noise, when compared to the manually created trajectory D9M, for example, ${\hat{s}}_{0}=29.8$ at 100 kg.

More details are shown in Table 7. For the error correction parameters $\left\{k_{0},k_{1}\right\}$ theoretical values, and therefore deviations, are not obtainable. Differences between the methods partly result from different experimental setups, as static and dynamic methods were measured separately, where load positioning may vary slightly between the successive runs. N-optimal quasi-static results are extracted from the full quasi-static measurement by deleting 450 non-optimal poses.

It should be noted that a correct physical representation of the parameters is not the main focus of the identification procedure. In fact, all model parameters together must allow an accurate force measurement after calibration. Details on accuracy and precision in practise for quasi-static, dynamic and process force measurements with the end-effector setup are presented in [1]. All identification methods show acceptable accuracy and are suitable for the desired fast re-calibration of the measurement model. Hereby, dynamic measurement allows the identification of all parameters in a very fast measuring process (12 s), whereas the slightly slower quasi-static measurement (81 s) shows less noise at load conditions but does not include the inertia.

## 9. Summary

Structure-integrated force measurement is an accurate and cost-efficient alternative to classic force measurement in 6 DoF. To compensate for machine internal influences, such as dynamics, a measurement model is essential. Depending on the measuring task, classic solutions using FT sensors at the end-effector also require or benefit from a measurement model. However, every model needs a parametrisation that, in this case, both determines the measuring accuracy and, furthermore, can change during machine usage. Following modelling and model analysis, two methods are presented to identify these measurement model parameters, which run in a fast and easy procedure, in an operational machine state and do not require any additional knowledge or setup-efforts from the machine user. Where quasi-static identification measurement is based on data acquisition in stationary conditions at several poses, dynamic identification measurement is based on highly accelerated movements while taking samples continuously. Both methods are applied to the hexapod-based end-effector with integrated force sensors and are optimised to find the most significant measuring poses and the best most exciting trajectory, respectively. By the application and re-identification of reference payloads both methods are validated and compared. In conclusion, all of the presented procedures allow for an accurate determination of the model parameters with relative deviations to the theoretical values that are, in most cases, significantly smaller than the maximum error of −% (${}^{P}z_{C}$ Q5N, 100 kg), where the quasi-static method shows fewer uncertainties at load conditions (${\hat{s}}_{0}=2.3$ Q5M vs. ${\hat{s}}_{0}=46.6$ D9N, 100 kg) and the dynamic method is faster (12 s D9N and 20 s D9M vs. 81 s Q5N and 21 min Q5M) and allows inertia identification.

## Figures and Tables

**Table 1:** Overview of approach, variations and methods.

Step/Approach		Quasi-Static Identification	Dynamic Identification
1.	Identification model (Section 4)	Models 0, 1, 2, 3, 4, 5	Models 6, 7, 8, 9
2.	Identifiability analysis (Section 5)	Measurement simulation, Pearson correlation, QR decomposition	
3.	Excitation generation	*Manually*: Brute force *Algorithmically*: Data reduction with 4 criteria (Section 6)	*Manually*: G-Code *Algorithmically*: Optimal Fourier-series with 4 criteria, 7 orders, 3 solvers (Section 7)
4.	Validation (Section 8)	Identification of reference payload for selected models and excitations	
		Q5M: Quasi-static, model 5, manual trajectory Q5N: N-optimal trajectory	D9M: Dynamic, model 9, manual trajectory D9N: N-optimal trajectory

**Table 2:** Identification modelling approaches with parameters, No.: Number of parameters, Identification results are presented in Table 3; In squares: selected models for excitation optimisation.

Model & Parameters		No.	Comment
Quasi-static Identification			
0	$m_{P}^{*}$, ${}^{P}x_{C}^{*}$, $f_{q0}$	10	No error correction.
1	$m_{P}^{*}$, ${}^{P}x_{C}^{*}$, $f_{q0}$, $K_{1}$	22	XY error correction, parameters not minimal.
2	$m_{P}^{*}$, ${}^{P}x_{C}^{*}$, $f_{q0}$, $K_{2}$	12	XY error correction, parameters minimal.
3	$m_{P}$, ${}^{P}x_{C}$, $f_{q0}$, $K_{2}$, $K_{3}$	12 + x	Not identifiable in any expression.
4	$m_{P}$, ${}^{P}x_{C}$, $f_{q0}$, $K_{2}$, $K_{4}$	30	Full error correction: correct $m_{P}$ and ${}^{P}x_{C}$.
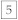	$m_{P}$, ${}^{P}x_{C}$, $f_{q0}$, $K_{2}$, $K_{5}$	14	Full error correction: correct $m_{P}$ and ${}^{P}x_{C}$.
Dynamic Identification			
6	$m_{P}$, ${}^{P}x_{C}$, $f_{q0}$, ${}^{P}I_{P}$	16	No error correction.
7	$m_{P}$, ${}^{P}x_{C}$, $f_{q0}$, ${}^{P}I_{P}$, $K_{2}$	18	XY error correction.
8	$m_{P}$, ${}^{P}x_{C}$, $f_{q0}$, ${}^{P}I_{P}$, $K_{2}$, $K_{4}$	36	Full error correction, parameters not minimal
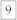	$m_{P}$, ${}^{P}x_{C}$, $f_{q0}$, ${}^{P}I_{P}$, $K_{2}$, $K_{5}$	20	Full error correction.

**Table 3:** Identification results for different modelling approaches, as listed in Table 2. Model 3 is not identifiable. Quasi-static results are based on measurements with 480 poses (Section 6). Dynamic results are based on manually chosen trajectory (Section 7). CAD: A-priori values from geometric design. In squares: selected models for excitation optimisation.

Parameter		CAD	Quasi-Static Identification					Dynamic Identification			
			**Model 0**	**Model 1**	**Model 2**	**Model 4**	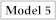	**Model 6**	**Model 7**	**Model 8**	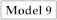
${\hat{s}}_{0}$	-	-	5.24	1.54	1.54	0.97	1.28	6.80	5.61	5.09	5.11
$m_{P}$	[kg]	33	30.36	30.36	30.36	33.78	33.78	32.13	31.51	31.83	31.83
${}^{P}x_{C}$	[mm]	0	0.52	0.52	0.52	−0.73	0.46	0.58	−0.17	0.14	0.01
		0	0.55	0.55	0.55	0.10	0.50	0.33	−0.07	−0.20	−0.32
		128	74.91	74.91	74.91	108.93	108.93	104.16	97.73	105.0	105.0
$f_{q0}$	[N]	-	−60.8	−60.8	−60.8	−54.9	−53.9	−53.4	−55.0	−54.3	−54.4
		-	38.6	38.6	38.6	46.1	45.5	42.0	40.7	41.4	41.4
		-	324.4	324.4	324.4	331.0	331.2	329.5	328.0	328.5	328.4
		-	−142.6	−142.6	−142.6	−135.2	−135.8	−137.9	−138.3	−138.0	−137.8
		-	−18.1	−18.1	−18.1	−10.2	−11.3	−14.6	−15.5	−14.6	−14.5
		-	−21.1	−21.1	−21.1	−15.5	−14.3	−18.7	−20.6	−20.0	−20.1
${}^{P}I_{P,C}$	[kg·m${}^{2}$]	1.65	-	-	-	-	-	0.94	1.02	1.25	1.26
		1.65	-	-	-	-	-	0.87	0.99	1.22	1.21
		1.51	-	-	-	-	-	0.92	0.92	0.88	0.91
$k_{0}$	[N/mm]	-	-	$K_{2}^{6\times 2}$	21.0	21.0	21.0	-	25.54	24.0	24.0
$k_{1}$	[N/mm]	-	-		−8.9	−8.9	−8.9	-	−9.65	−8.47	−8.46
$k_{2}$	[N/rad]	-	-	-	-	$K_{4}^{6\times 3}$	65.5	-	-	$K_{4}^{6\times 3}$	37.39
$k_{3}$	[N/rad]	-	-	-	-			26.9	-	-	28.2

**Table 5:** Optimisation of quasi-static identification procedure with model 5 through a reduction of the number of poses to 30 starting from algorithmically generated 480 poses; last row: normal probability density plots for the parameters.

Parameter		No Opt. (Q5M)		A Opt. (Q5A)		D Opt. (Q5D)		K Opt. (Q5K)		N Opt. (Q5N)	
Poses		480		30		30		30		30	
Min. poses		-		3		4		4		3	
${\hat{s}}_{0}$	-	1.28		1.83		1.77		1.39		1.81	
		$\hat{u}$	${\hat{s}}_{j}\left(\hat{u}\right)$	$\hat{u}$	${\hat{s}}_{j}\left(\hat{u}\right)$	$\hat{u}$	${\hat{s}}_{j}\left(\hat{u}\right)$	$\hat{u}$	${\hat{s}}_{j}\left(\hat{u}\right)$	$\hat{u}$	${\hat{s}}_{j}\left(\hat{u}\right)$
$m_{P}$	[kg]	33.78	0.22	33.02	0.94	33.66	0.90	33.69	1.00	32.96	0.92
${}^{P}x_{C}$	[mm]	0.46	0.19	1.02	0.93	0.89	0.87	1.94	0.75	1.16	0.90
		0.50	0.19	0.49	0.87	0.29	0.82	−0.25	0.78	0.38	0.89
		108.93	1.34	105.93	5.14	111.36	4.87	110.01	5.38	106.75	5.08
$f_{q0}$	[N]	−53.94	0.49	−54.67	2.03	−53.63	1.96	−53.18	2.05	−54.78	1.99
		45.45	0.49	43.52	2.02	44.84	1.95	44.80	2.06	43.45	2.00
		331.17	0.49	329.90	2.02	331.11	1.96	329.59	2.12	329.32	1.99
		−135.76	0.49	−137.03	2.02	−135.64	1.96	−135.05	2.10	−136.56	1.99
		−11.33	0.49	−12.86	2.03	−11.67	1.95	−12.37	2.10	−13.47	2.02
		−14.26	0.49	−15.23	2.04	−13.93	1.96	−12.90	2.11	−15.01	2.02
$k_{0}$	[N/mm]	20.99	0.27	20.82	1.19	20.97	1.14	19.95	1.19	20.98	1.22
$k_{1}$	[N/mm]	−8.90	0.05	−8.74	0.22	−8.99	0.21	−8.56	0.22	−8.78	0.22
$k_{2}$	[N/rad]	65.50	4.28	50.98	17.70	61.82	17.08	66.64	18.93	49.46	17.45
$k_{3}$	[N/rad]	26.87	0.86	23.33	3.21	27.51	3.10	28.19	3.43	23.45	3.16
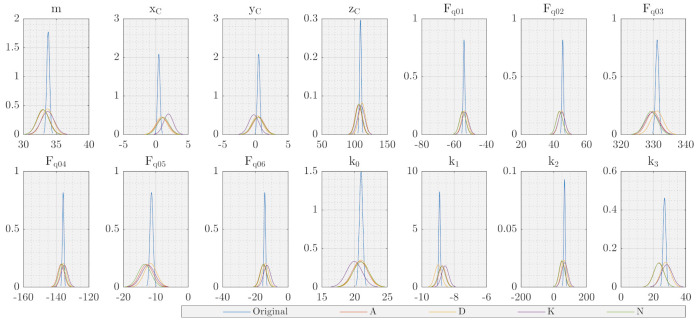											

**Table 6:** Identification results using model 9 for dynamic measurements with manually created and optimised Fourier series trajectory, compare Figure 5.

Parameter		Dyn. Manual Traj. (D9M)		Dyn. N-optimal Traj. (D9N)	
Poses		19,820		11,530	
${\hat{s}}_{0}$	-	5.11		8.59	
		$\hat{u}$	${\hat{s}}_{j}\left(\hat{u}\right)$	$\hat{u}$	${\hat{s}}_{j}\left(\hat{u}\right)$
$m_{P}$	[kg]	31.83	0.02	31.88	0.04
${}^{P}x_{C}$	[mm]	0.01	0.13	1.60	0.25
		−0.32	0.12	−1.09	0.25
		105.05	0.12	107.40	0.29
$f_{q0}$	[N]	−54.41	0.13	−52.80	0.27
		41.37	0.13	41.73	0.27
		328.38	0.13	328.63	0.27
		−137.75	0.13	−137.58	0.27
		−14.46	0.14	−13.04	0.27
		−20.11	0.14	−16.63	0.27
${}^{P}I_{PC},$	[kg·m${}^{2}$]	1.26	0.005	1.38	0.03
		1.21	0.005	1.18	0.02
		0.91	0.005	1.02	0.02
$k_{0}$	[N/mm]	24.05	0.24	30.75	0.36
$k_{1}$	[N/mm]	−8.46	0.04	−9.49	0.07
$k_{2}$	[N/rad]	37.38	0.89	93.00	1.13
$k_{3}$	[N/rad]	28.27	0.19	27.78	0.27

**Table 7:** Selected identified parameters and relative differences to the theoretical physical values of benchmark payloads for quasi-static and dynamic approaches; characteristics of each applied mass step: m=19.93kg, h=60mm, $x_{C}=\left\{0,10.3,31.1\right\}\;\mathrm{mm}$, $I_{\mathrm{zz}}=0.1536\;{\mathrm{kgm}}^{2}$.

Param. / Load		0 kg	20 kg		40 kg		60 kg		80 kg		100 kg		
${\hat{s}}_{0}$	-	1.28	1.37		1.48		1.65		1.94		2.30		Q5M: Quasi-static ident. model 5 with 480 poses
$m_{P}$	[kg]	33.78	53.48	(−0.4%)	72.92	(−1.0%)	91.98	(−1.7%)	111.05	(−2.2%)	130.00	(−2.6%)	
${}^{P}z_{C}$	[mm]	108.93	98.54	(−0.1%)	110.65	(−0.1%)	129.35	(−1.2%)	152.92	(−1.4%)	178.43	(−1.5%)	
$f_{q02}$	[N]	331.17	330.35	(−0.2%)	328.74	(−0.7%)	327.28	(−1.2%)	325.78	(−1.6%)	323.26	(−2.4%)	
$k_{0}$	[N/mm]	20.99	22.36		23.83		25.58		27.05		28.44		
$k_{1}$	[N/mm]	−8.90	−9.54		−10.18		−11.00		−11.66		−12.36		
${\hat{s}}_{0}$	-	1.81	1.93		2.07		2.33		2.74		3.14		Q5N: Quasi-stat. ident. model 5 with 30 N-optimal poses
$m_{P}$	[kg]	32.96	52.89	(−0.0%)	72.33	(−0.7%)	91.22	(−1.7%)	110.57	(−1.9%)	129.19	(−2.5%)	
${}^{P}z_{C}$	[mm]	106.75	95.34	(−1.8%)	107.43	(−2.1%)	126.41	(−3.1%)	148.68	(−3.9%)	174.02	(−3.9%)	
$f_{q02}$	[N]	329.32	328.46	(−0.3%)	327.04	(−0.7%)	325.08	(−1.3%)	324.42	(−1.5%)	321.19	(−2.5%)	
$k_{0}$	[N/mm]	20.98	21.87		23.02		25.21		26.62		28.12		
$k_{1}$	[N/mm]	−8.78	−9.43		−10.09		−10.92		−11.65		−12.38		
${\hat{s}}_{0}$	-	5.11	7.48		10.60		14.57		20.70		29.75		D9M: Dyn. ident. model 9 with manually designed trajectory
$m_{P}$	[kg]	31.83	50.98	(−1.5%)	69.93	(−2.5%)	89.13	(−2.7%)	108.46	(−2.7%)	128.19	(−2.5%)	
${}^{P}z_{C}$	[mm]	105.06	95.68	(−0.2%)	108.90	(−0.2%)	130.02	(−0.1%)	154.78	(+0.0%)	182.18	(+0.5%)	
${}^{P}I_{P.\mathrm{Czz}}$	[kgm${}^{2}$]	0.92	1.06	(−0.6%)	1.21	(−1.1%)	1.36	(−1.5%)	1.50	(−1.8%)	1.65	(−1.7%)	
$f_{q02}$	[N]	328.27	328.22	(−0.0%)	324.73	(−1.1%)	323.82	(−1.4%)	322.78	(−1.7%)	320.14	(−2.5%)	
$k_{0}$	[N/mm]	24.03	28.15		31.79		34.60		35.06		33.99		
$k_{1}$	[N/mm]	−8.44	−9.24		−10.04		−10.80		−11.28		−11.37		
${\hat{s}}_{0}$	-	8.59	12.27		17.69		25.03		34.35		46.62		D9N: Dyn. ident. model 9 with periodic N-optimal 3rd order trajectory
$m_{P}$	[kg]	31.88	51.14	(−1.2%)	70.25	(−2.0%)	90.19	(−1.6%)	110.09	(−1.4%)	129.98	(−1.2%)	
${}^{P}z_{C}$	[mm]	107.40	97.16	(−0.1%)	110.24	(+0.1%)	131.00	(+0.1%)	155.78	(+0.3%)	182.59	(+0.4%)	
${}^{P}I_{P.\mathrm{Czz}}$	[kg·m${}^{2}$]	1.02	1.17	(−0.1%)	1.34	(+1.6%)	1.50	(+1.5%)	1.65	(+1.5%)	1.82	(+2.2%)	
$f_{q02}$	[N]	328.63	329.19	(+0.2%)	325.75	(−0.9%)	327.05	(−0.5%)	326.43	(−0.7%)	325.17	(−1.1%)	
$k_{0}$	[N/mm]	30.75	38.10		44.56		47.87		50.04		52.85		
$k_{1}$	[N/mm]	−9.49	−10.43		−11.43		−12.22		−12.53		−13.09		

## Data Availability

The data presented in this study are available on request from the corresponding author.

## Abbreviations

The following abbreviations and symbols are used in this manuscript:

${}^{A}{\left(\cdot \right)}_{B}$	Coordinate vector/tensor: top left reference frame, bottom right body frame
$\hat{\left(\cdot \right)}$, $\overset{ˇ}{\left(\cdot \right)}$	Estimated values, and measured values
$\left(\underline{\cdot }\right)$, $\left(\bar{\cdot }\right)$	Spatial vector (6×1), and vectorized matrix
${\left(\cdot \right)}^{-T}$	Transposed inverse
$f_{q}$, $f_{q0}$, $f_{K}$, $f_{q}^{*}$	Measured forces, sensor bias, correction values, and processed sensor forces
${\underline{f}}_{\mathrm{Ext}}$, ${\underline{f}}_{\mathrm{Dyn}}$, ${\underline{f}}_{G}$	External, Dynamic, and Gravitational spatial forces and moments
$J_{P}$, $J_{\mathrm{Hex}}\left(\underline{x}\right)$	Jacobian of the sensor framework and Jacobian of the hexapod kinematic
$m_{P}$, ${}^{P}x_{C}$, ${}^{P}I_{P}$	Mass, centre of gravity, and inertia of the platform
q, $\dot{q}$, $\ddot{q}$	Position, velocity, and acceleration of the drives
$\underline{x}$, ${}^{B}o_{P}$, ${}^{B}R_{P}$	Pose, position, and orientation of the TCP
$\underline{v}$, ${}^{B}{\dot{o}}_{P}$, ${}^{B}\omega_{P}$	Velocity, translational velocity, and angular velocity of the TCP
$\dot{\underline{v}}$, ${}^{B}{\ddot{o}}_{P}$, ${}^{B}{\dot{\omega }}_{P}$	Acceleration, translational acceleration, and angular acceleration of the TCP
$M_{P}$, $C_{P}$, $g_{P}$	Mass matrix, matrix of centrifugal and Coriolis terms, gravitational terms
A, u	Identification model, vector of unknowns
m, u, n	Number of: measurements, unknowns, forces per measurement
H, W, $\tilde{A}$	Scaling matrix, weighting matrix, and scaled model
${\hat{s}}_{0}$, ${\hat{s}}_{j}$, $\hat{M}$	Global standard deviation, local standard deviations, and covariance matrix
Q5M	Quasi-static approach Model 5, manually generated poses
Q5[A,D,K,N]	Quasi-static approach Model 5, A, D, K or N optimal arithm. gen. poses
D9M	Dynamic approach Model 9, manually generated trajectory
D9[A,D,K,N]	Dynamic approach Model 9, A, D, K or N optimal arithm. gen. trajectory
CAD	Computer-Aided Design
CNC	Computerized Numerical Control
DoF	Degree of Freedom
FK	Forward Kinematic
TCP	Tool Center Point
